# MicroRNA-25 Protects Smooth Muscle Cells against Corticosterone-Induced Apoptosis

**DOI:** 10.1155/2019/2691514

**Published:** 2019-03-12

**Authors:** Bin Zhang, Gaoxing Zhang, Tianlu Wei, Zhen Yang, Wenfeng Tan, Ziqing Mo, Jinxue Liu, Dong Li, Yidong Wei, Lukun Zhang, Keith A. Webster, Jianqin Wei

**Affiliations:** ^1^Department of Cardiovascular Disease, The Jiangmen Central Hospital, Jiangmen 529030, China; ^2^Clinical Experimental Center, Jiangmen Central Hospital, Affiliated Jiangmen Hospital of Sun Yat-Sen University, Jiangmen 529030, China; ^3^Department of Medicine, The First Affiliated Hospital of Guangxi University of Chinese Medicine, Nanning 530023, China; ^4^Department of Cardiovascular, Sun Yat-Sen University, Guangzhou 510080, China; ^5^Department of Intensive Care Unit, The Jiangmen Central Hospital, Jiangmen 529030, China; ^6^Youjiang Medical University for Nationalities, Chengxiang Rd, Baise, Guangxi 533000, China; ^7^Department of Infection, Third People's Hospital of Shenzhen, 29 Bulan Road, Shenzhen 518112, China; ^8^Department of Molecular and Cellular Pharmacology and the Vascular Biology Institute, Miller School of Medicine, University of Miami, FL 33136, USA; ^9^Department of Medicine, Division of Cardiology, Miller School of Medicine, University of Miami, FL 33136, USA

## Abstract

**Background and Aims:**

Vascular smooth muscle cells (VSMCs) are central components of atherosclerotic plaque. Loss of VSMCs through apoptotic cell death can cause fibrous cap thinning, necrotic core formation, and calcification that may destabilize plaque. Elevated glucocorticoid levels caused by psychological stress promote VSMC apoptosis and can exacerbate atherosclerosis in mice and humans. Changes in the levels of antiapoptosis microRNA-25 (miR-25) have been linked with heart disease, inflammation, VSMC phenotype, oxidative stress, and apoptosis. Here, we investigated the pathways and mechanisms of glucocorticoid-induced apoptosis of mouse VSMCs and the protective role of miR-25.

**Methods:**

Primary mouse VSMCs were cultured +/- corticosterone for 48 h. Apoptosis, ROS, apoptotic protein activities, miR-25, MOAP1, a miR-25 target, and p70S6 kinase were quantified at intervals. The roles of miR-25 were assessed by treating cells with lenti-pre-miR-25 and anti-miR-25.

**Results:**

VSMC apoptosis, caspase-3 activity, and Bax were increased by corticosterone, and cell death was paralleled by marked loss of miR-25. Protection was conferred by pre-miR-25 and exacerbated by anti-miR-25. Pre-miR-25 conferred reduced expression of the proapoptotic protein MOAP1, and the protective effects of pre-miR-25 were abrogated by overexpressing MOAP1. The antiapoptotic effects of miR-25 were paralleled by inhibition of the p70S6K pathway, a convergence target for the survival signaling pathways, and protection by pre-miR-25 was abrogated by the p70S6k inhibitor rapamycin.

**Conclusions:**

MicroRNA-25 blocks corticosterone-induced VSMC apoptosis by targeting MOAP1 and the p70S6k pathway. Therapeutic manipulation of miR-25 may reduce atherosclerosis and unstable plaque formation associated with chronic stress.

## 1. Introduction

Glucocorticoids are anti-inflammatory, immune-suppressive hormones that contribute importantly to the regulation of glucose metabolism and blood homeostasis. They are released from the adrenal gland at various stages of development and in adults especially during occupational stress exposure. Because of their anti-inflammation properties, glucocorticoids have clinical applications in the treatment of pain associated with inflammation and a range of conditions that involve overactive immune systems such as transplantation, allergy, and autoimmune disease. Glucocorticoids are also used to enhance the therapeutic effects of diuretics and natriuretic peptides in patients with advanced heart failure. Obviously, more chronic clinical use of glucocorticoids are limited by their potentially serious adverse side effects [1–3].

Epidemiological studies support roles for elevated glucocorticoid levels in exacerbating coronary artery disease risk and progression associated with psychological stress, especially occupational stress in humans and environmental stress in rodents [4–7]. Cortisol is the major glucocorticoid of humans, whereas the rodent equivalent is corticosterone. The relationships between environmental stress, glucocorticoids, and atherosclerosis; their molecular and cellular signaling pathways; and their mechanisms of interaction are poorly understood.

VSMCs play pivotal roles in atherogenesis including initiation, progression, and stability of plaques [8]. Phenotypic switching, abnormal proliferation, and turnover of VSMCs are components of plaque growth and expansion as well as stability of mature plaques. VSMC apoptosis in particular can influence plaque stability by causing fibrous cap thinning, necrotic core formation, and calcification. VSMC apoptosis is induced by proinflammatory cytokines, oxidized low-density lipoprotein, and mechanical injury [9, 10]. Glucocorticoid-induced VSMC apoptosis has been recognized for 2 decades, although the targets and mechanisms are still unclear.

Multiple noncoding RNAs known as microRNAs (miRs) have been reported to control VSMC turnover and apoptosis [11–13]. For example, miR-21, miR-26a, miR-29b, and miR-126 have been variously reported to regulate the balance between VSMC apoptosis and proliferation in different vascular tissues while miR-143 and miR-145 confer growth regulation by determining phenotypic switching [11–13]. Targets for such miRs include growth factor pathway intermediates, ROS production, transcription factors, cell cycle, and/or apoptosis control points.

miR-25 is part of the miR-106b/25 cluster located on chromosome 7q22 of the host maintenance protein 7 (MCM7), a gene that is highly conserved in vertebrates [14]. MCM7 is a component of the MCM2-7 complex of DNA helicases that are required for the initiation of DNA replication in eukaryotic cells [14–17]. miR-25 has 1163 predicted target mRNA transcripts (TargetScanHuman version 7.1) and can target mRNAs involved in DNA damage, cell cycle regulation, cell proliferation, migration, and differentiation under physiological as well as pathological conditions (reviewed in [18]). It has been implicated in the development and spread of many tumor types and shown to confer important regulation of apoptosis, autophagy, oxidative stress, inflammation, and calcium handling. As such, miR-25 is implicated in the pathogenesis of multiple conditions including but not limited to acute myocardial infarction, heart failure, diabetes, nephropathies, cerebral ischemia/reperfusion injury, neurodegenerative diseases, schizophrenia, and multiple sclerosis (reviewed in [18]). Direct cell signaling pathway targets for miR-25 include FOXO3a, SOCS4, SOX4, RGS3, NOX4, Cyclin E2, Smad7, PKC, Krüppel-like factor 4 (KLF4), ERK, PTEN, and WNT/*β*-catenin [19–25]. In cancer cells, miR-25 was shown to target the proapoptotic protein MOAP1 while its antiapoptosis protective effects in other cell types have been linked with activation of the PTEN/Akt and AMPK signaling pathways [26]. Other studies documented protective roles for miR-25 in osteoblast responses to dexamethasone [27].

Here, we sought to investigate roles for miR-25 in the responses of VSMCs to treatment with the glucocorticoid corticosterone. We show that the increased rate of apoptosis of VSMCs caused by corticosterone is mediated by ROS, downregulation of miR-25, and enhanced expression of proapoptotic protein MOAP1. Protection was afforded by overexpression of miR-25 and exacerbated by anti-miR-25 or MOAP1 overexpression. Corticosterone activated the p70 ribosomal protein S6 kinase 1 (S6K1) pathway in a miR-25-dependent manner, and corticosterone-dependent apoptosis was significantly alleviated by rapamycin, a selective S6K1 inhibitor.

## 2. Materials and Methods

### 2.1. Reagents

Antibodies were obtained from the following vendors: anti-caspase-3, cleaved caspase-3, BCL2, GAPDH, and pp-70S6K from Cell Signaling Technology; anti-Bax from Santa Cruz; anti-MOAP1, corticosterone, and rapamycin from Sigma; and human pre-microRNA expression construct lenti-pre-miR-25 and anti-miR-25 from System Biosciences LLC.

### 2.2. Smooth Muscle Cell Culture and Treatment

C57BL/6 mouse primary aortic smooth muscle cells (Cell Biologics) were cultured as previously described [28–31]. Corticosterone and rapamycin were added to the culture media at 30 ng/mL and 25 nM/mL, respectively. Cells were infected with lentivirus as described previously [32].

### 2.3. Measurement of ROS Productions

ROS were measured using an OxiSelect ROS assay kit from Cell Biolabs, San Diego, as described by the manufacturer.

### 2.4. Cell Apoptosis Assay

Apoptosis was analyzed by TUNEL assay using a Click-iT® Plus TUNEL Assay Kit (Life Technologies Inc.) and was performed according to the manufacturer's instructions. TUNEL-positive nuclei of six nonoverlapping fields per coverslip were counted by a researcher blinded to treatment, and these counts were converted to percentages by comparing the TUNEL-positive counts to the total number of cell nuclei as determined by DAPI counterstaining: TUNEL − positive ratio = (number of red nucleinumber of blu>e nuclei) × 100%.

### 2.5. Luciferase Reporter Assay

Luciferase assays were performed as previously described [33]. Forty-eight hours after transfection, cell extracts were assayed for luciferase activity using a Luc-Pair miR luciferase assay kit (GeneCopoeia). Relative reporter activities were expressed as luminescence units normalized to Renilla luciferase activity. Luminescence was quantitated using a multimode microplate reader (BMG Labtech).

### 2.6. Quantitative RT-PCR

Total RNA was isolated from myocardial tissue using TRIzol reagent and analyzed by qRT-PCR using TaqMan probes (Applied Biosystems, Foster City, CA, USA) as described previously [34–37]. The miR-25 primers were Fw 5′-CGG CGG CAT TGC ACT TGG TCT C-3′ and Rv 5′-GTG CAG GGT CCG AGG T-3′. Primers for detection of MOAP1 mRNA were Fw TGA ACC CTC GGA AAG CG and Rv TTC TCA TCC CTC CTG AAC ATC. miRNA quantifications are presented as ratio of miRNA/5S-rRNA.

### 2.7. Western Blot

Our procedures for western blotting are described in detail elsewhere [38]. Briefly, 40 *μ*g of protein was separated by 12% SDS-PAGE and transferred onto nitrocellulose, blocked with 5% fat-free skim milk in TBS containing 0.05% Tween 20 (1 h at room temperature), and incubated with antibodies overnight at 4°C. Protein bands were visualized by ECL detection reagent.

### 2.8. Statistical Analysis

Data are expressed as mean ± S.E.M. Statistical comparisons were performed using ANOVA followed by paired, one-tailed *t*-test, using InStat software for Macintosh (GraphPad Software Inc.).

## 3. Results

### 3.1. Induction of ROS and Apoptosis in VSMCs by Corticosterone

**Figure 1:**
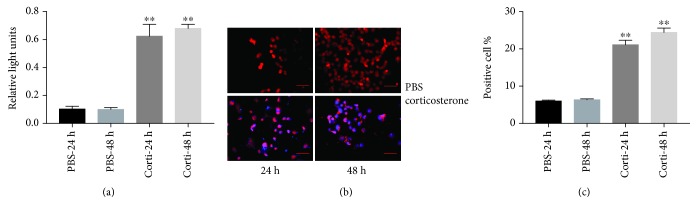
Increased ROS and apoptosis by corticosterone treatment. VSMCs were treated with corticosterone. Reactive oxygen species (ROS) were measured using an OxiSelect ROS assay kit and expressed as relative light units (RLU) (a). Apoptosis was detected by TUNEL assay (b, c). Results are expressed as mean ± S.E.M.^∗∗^*p* < 0.01. PBS-24 and PBS-48 h: VSMCs were treated with PBS 24 and 48 hours; Corti-24 h and Corti-48 h: VSMCs were treated with corticosterone as indicated for 24 and 48 hours.

**Figure 2:**
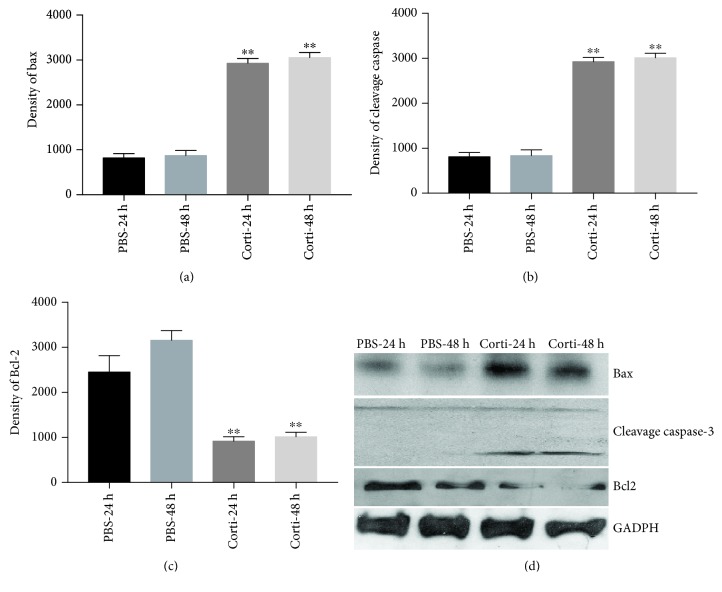
Corticosterone induces expression of apoptosis-associated proteins in VSMCs. VSMCs were treated with corticosterone. Western blot analyses show expression of Bax (a), cleaved caspase-3 (b), and Bcl-2 (c); bands on western blot depict Bax, cleaved caspase-3, Bcl-2, and GAPDH. Results are expressed as mean ± S.E.M.^∗∗^*p* < 0.01. PBS-24 and PBS-48 h: VSMCs were treated with PBS for 24 and 48 hours; Corti-24 h and Corti-48 h: VSMCs were treated with corticosterone for 24 and 48 hours.

As shown in Figure 1(a), ROS production increased by 5.9- and 6.7-fold in VSMCs treated with corticosterone for 24 h and 48 h, respectively (both *p* < 0.01). Apoptotic indices increased in parallel by 21.34% and 24.67% in the corticosterone treatment groups compared with 6.24% and 6.56% in controls (*p* < 0.01), determined by TUNEL staining (Figures 1(b) and 1(c)). Western analyses confirmed significantly increased levels of Bax (2.4- and 3.5-fold; *p* < 0.01), cleaved caspase-3 (2.6- and 3.5-fold; *p* < 0.01), and decreased Bcl-2 (2.6- and 3-fold; *p* < 0.01), at each time point, shown in Figures 2(a)–2(d). These results confirm that corticosterone promotes apoptosis of VSMCs by the intrinsic, mitochondrial pathway.

### 3.2. Downregulation of miR-25 in VSMCs by Corticosterone Treatment

**Figure 3:**
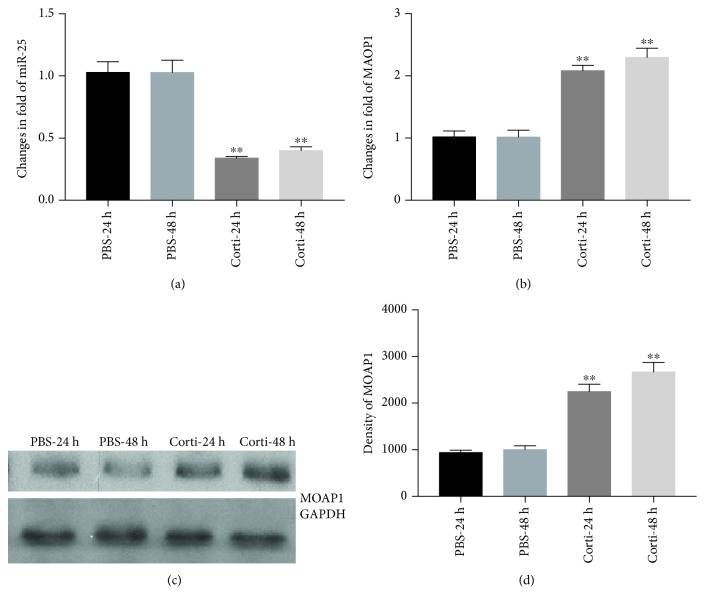
Downregulation of miR-25 and increased MOAP1 expression in VSMCs induced by corticosterone treatment. VSMCs were treated with corticosterone. MicroRNA-25 was measured by qRT-PCR and expressed as fold changes (a). Expressions of MOAP1 were detected by RT-PCR and western blot analysis (b, c, and d). Results are expressed as mean ± S.E.M.^∗∗^*p* < 0.01. PBS-24 and PBS-48 h: VSMCs were treated with PBS for 24 and 48 hours; Corti-24 h and Corti-48 h: VSMCs were treated with corticosterone for 24 and 48 hours.

MicroRNA-25 levels were quantified by qRT-PCR after culture of VSMCs in the presence and absence of corticosterone for 24 or 48 hours as described in Materials and Methods. We found that miR-25 levels were decreased by 63.2% and 59% (both *p* < 0.01) after 24 and 48 hours, respectively, of corticosterone treatment (Figure 3(a)).

### 3.3. Corticosterone Induces Expression of MOAP1 in VSMCs

MOAP1 mRNA expression in VSMCs groups ± corticosterone as indicated above was determined using qRT-PCR. As expected, MOAP1 levels increased significantly by 2.1- and 2.3-fold (both *p* < 0.01) after 24 h and 48 h of treatment, as shown in Figure 3(b). Western blots further revealed that MOAP1 protein increased in parallel by 2.3- and 2.6-fold (both *p* < 0.01) at the 24 h and 48 h treatment times, as shown in Figures 3(c) and 3(d). These results demonstrate that corticosterone induced increased expression of MOAP1 in VSMCs in parallel with the apoptotic pathway described in Figure 1.

### 3.4. MOAP1 Is a Target of miR-25

**Figure 4:**
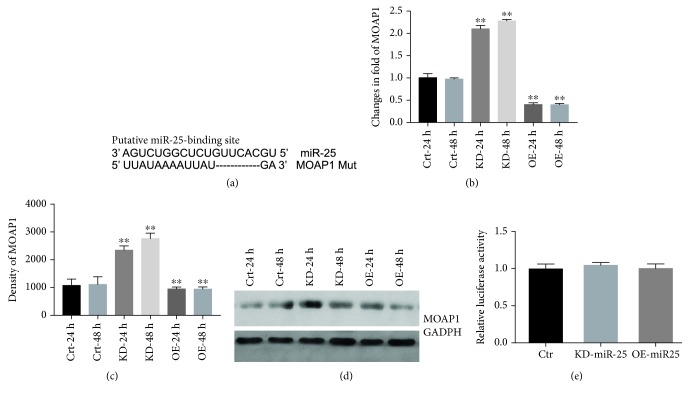
MOAP1 is a direct target of miR-25. MicroRNA-25 seed sequence and its complementary binding site in MOAP1 3′-UTR are highlighted (a). VSMCs were untreated or transfected with pre-miR-25 or antagomiR-25 for 24 h or 48 h. Expression of MOAP1 mRNA was determined using qRT-PCR and western blot (b, c, and d). Luciferase activities of constructs containing the mutant 3′-UTR segment of MOAP1 (e). Results are expressed as mean ± S.E.M.^∗∗^*p* < 0.01. PBS-24 and PBS-48 h: VSMCs were treated with PBS for 24 and 48 hours. Corti-24 h and Corti-48 h: VSMCs were treated with corticosterone for 24 and 48 hours; Crt-24 h, Crt-48 h, KD-24 h, KD-48 h, OE-24 h, and OE-48 h: VSMCs were infected with control vector, pre-miR-25, or antagomiR-25 for 24 and 48 hours; Crt: control vector; KD-miR-25: antagomiR-25; and OE-miR-25: pre-miR-25.

It was reported recently that miR-25 can target MOAP1 mRNA in lung cancer cells [26]. Our studies confirmed the presence of an RNA-binding site for miR-25 in the 3′-UTR of the MOAP1 gene (Figure 4(a)) and suggest that the MOAP1 transcript is a direct target of miR-25. To test for functional regulation of apoptosis by miR-25 through a MOAP1 target, we infected VSMCs with lentiviral vectors expressing pre-miR-25 or anti-miR-25. Infection with pre-miR-25 caused a significant decrease in MOAP1 expression at both mRNA and protein levels, whereas transfection with antagomiR-25 caused a marked increase, consistent with miR-25 targeting of MOAP1 mRNA (Figures 4(b)–4(d)). Also consistent with a miR-25-MOAP1 interaction at the 3′-UTR, we found that a luciferase reporter construct with a mutant MOAP1 3′-UTR was not responsive to pre-miR or anti-miR-25 coinfection (Figure 4(e)). These results suggest that MOAP1 is a direct target of miR-25 in VSMCs consistent with previous work on lung cancer cells [26].

### 3.5. miR-25 Attenuates Oxidative Stress and Apoptosis by Targeting MOAP1 in VSMCs

**Figure 5:**
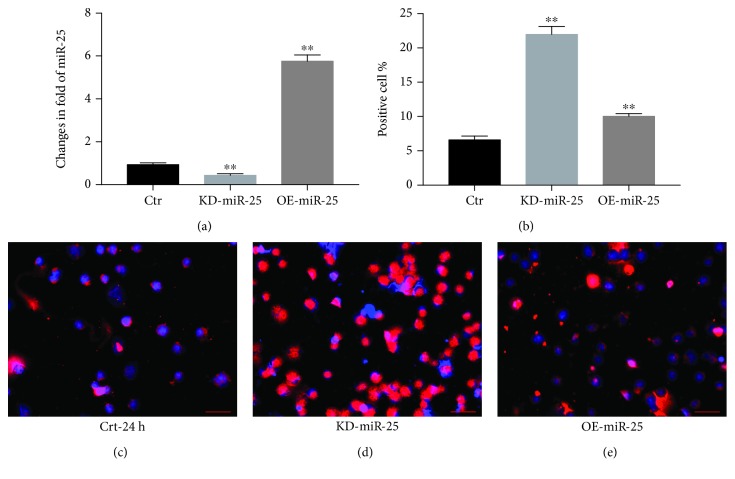
Attenuated apoptosis in VSMCs by miR-25. VSMCs were treated with corticosterone and transfected with pre-miR-25 or antagomiR-25 for 24 h. Expressions of miR-25 were measured by qRT-PCR, expressed as fold change relative to control (a); apoptosis was detected by TUNEL assay (b–e), expressed as positive cell %. Results are expressed as mean ± S.E.M.^∗∗^*p* < 0.01. Crt: control vector; KD-miR-25: antagomiR-25; and OE-miR-25: pre-miR-25.

**Figure 6:**
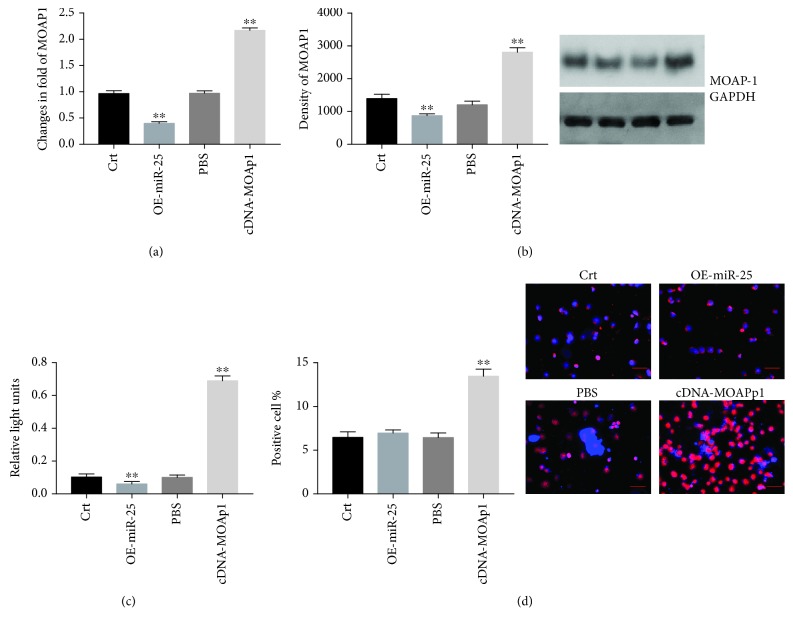
Attenuation of oxidative stress and apoptosis by miR-25-targeting of MOAP1. VSMCs were transfected with pre-miR-25 and pcDNA-MOAP1 for 24 h. Expressions of MOAP1 were measured by qRT-PCR and western blot (a, b); ROS were measured using an OxiSelect ROS assay kit (c); apoptosis was detected by TUNEL assay (d). Results are expressed as mean ± S.E.M.^∗∗^*p* < 0.01. Crt: control vector; OE-miR-25: pre-miR-25.

VSMCs were infected with lentiviral vectors expressing pre-miR-25 or antagomiR-25 and subjected to 24 h culture ± corticosterone as described in Figure 1. Expression of miR-25 was increased 5.8-fold by lenti-pre-miR-25 and decreased by 49.5% for anti-miR-25 (Figure 5(a)). TUNEL analysis revealed apoptotic indices of 6.78% in control cultures without treatments, 22.15% in the corticosterone plus anti-miR-25 group, and 10.23% in the corticosterone plus pre-miR-25 group (all *p* < 0.01) (Figure 5(b)). The results confirm that overexpression of miR-25 inhibits corticosterone-induced apoptosis in VSMCs. To confirm a role for MOAP1, VSMCs were transfected with plasmid pcDNA-MOAP1 and cultured ± corticosterone for 24 hours. We found that pre-miR-25 suppressed endogenous expression of MOAP1 in VSMCs and this was reversed in the pcDNA-MOAP1 treatment group (Figures 6(a) and 6(b)). Notably, pcDNA-MOAP1 significantly abrogated the effects of pre-miR-25 on both apoptosis and ROS production (Figures 6(c) and 6(d)). These results confirm that miR-25 protects VSMCs from corticosterone-induced cellular injury by targeting MOAP1.

### 3.6. miR-25 Inhibits p70S6K Activity Coincident with Decreased MOAP1 Expression in Corticosterone-Treated VSMCs

**Figure 7:**
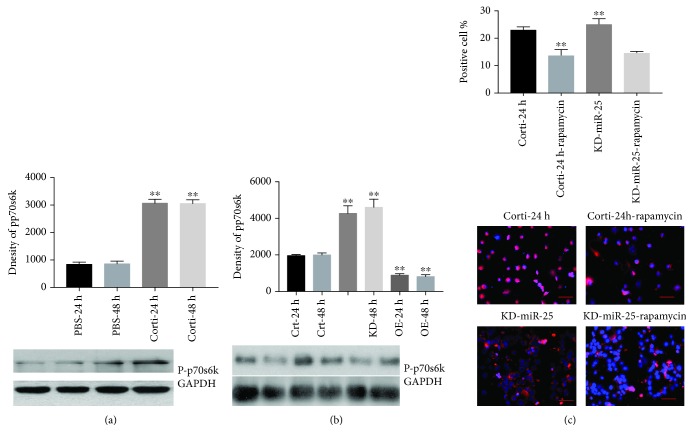
Crosstalk inhibition of p70S6K signaling by miR-25 in parallel with decreased MOAP1. VSMCs were treated with corticosterone and infected with pre-miR-25 or antagomiR-25. Expressions of pp70S6K were measured by western blot analysis (a, b). VSMCs were pretreated with rapamycin prior to treatment with corticosterone and infection with antagomiR-25. Results are expressed as mean ± S.E.M.^∗∗^*p* < 0.01. Crt-24 h and Crt-48 h: VSMCs were treated with PBS for 24 and 48 hours; Corti-24 h and Corti-48 h: VSMCs were treated with corticosterone for 24 and 48 hours; and KD-miR-25: antagomiR-25.

Crosstalk between the miR-25 and survival signaling pathways has been reported [11, 21, 26, 39]. Multiple such pathways converge on Akt and p70S6K. Therefore, we investigated a possible crosstalk between miR-25 and p70S6K in VSMCs treated with corticosterone (Figure 7). As expected, phosphorylated p70S6K (p-p70S6K) was increased in VSMCs by corticosterone (Figure 7(a)). When VSMCs were infected with pre-miR-25, corticosterone-induced p-p70S6K levels decreased while the reverse was observed in the antagomiR-25 cotreatment group (Figure 7(b)). To further investigate the relationship between miR-25 and p70S6K in this system, we treated VSMCs with rapamycin, a selective p70S6K inhibitor ± corticosterone, and antagomiR-25. As shown in Figure 7(c), apoptosis was differentially blocked by rapamycin. Corticosterone-induced apoptosis was partially blocked by rapamycin, whereas protection was significantly greater in the presence of both corticosterone and antagomiR-25 (Figure 7(c)). These results suggest that miR-25 can interfere with steps of p70S6K signaling in parallel with suppression of MOAP1 in corticosterone-treated VSMCs and this affects the degree of apoptosis. The regulation of p70S6K is likely a secondary consequence of the crosstalk either one or more of the multiple signaling pathways that are targets of miR-25. For example, miR-25 activates AMPK in dexamethasone-treated osteoblasts by direct targeting of PKC*ζ* and can activate PI3K/Akt signaling by targeting PTEN in liver cancer stem cells (new Refs. [21, 27]).

### 3.7. Discussion

Our studies show that physiological levels of corticosterone stimulate ROS production and apoptosis of cultured VSMCs. Corticosterone activated the mitochondrial intrinsic apoptotic pathway, and the effects were paralleled by markedly decreased miR-25 and activation of the MOAP1 and p70S6K pathways. Overexpression of miR-25 during exposure to corticosterone blocked apoptosis and inhibited both MOAP1 and p70S6K. The effects were abrogated by overexpression of MOAP1 or cotreatment with rapamycin. This is the first study to link glucocorticoid-activated apoptosis with miR-25, MOAP1, and the p70S6K signaling pathway. The results provide a mechanism for the role of psychological stress in exacerbating atherosclerosis and may provide new therapeutic avenues for its management.

Regulatory roles for multiple miRNAs in the progression of apoptosis, glucocorticoid actions, oxidative stress, and VSMC biology are well described [13, 19–25, 40–44]. Accumulating evidence indicates that miR-25 confers antiapoptotic functions in multiple cell and tissue types by targeting different genes and pathways. miR-25 was reported to inhibit apoptosis in lung cancer by targeting RGS3 and MOAP1 [22]. Roles for miR-25 in blocking apoptosis caused by oxidative stress of cardiac H92C and kidney tubule cells have been reported, and in one study, miR-25 was shown to target NOX4, thereby regulating NADPH oxidase activity [24, 25, 39]. The antiapoptosis roles, targets, and regulation of miR-25 are tissue-specific [24, 39, 45, 46]. Our work indicates that in SVMCs, glucocorticoid exposure mediates a downregulation of miR-25 that parallels increased ROS and this prevents the antiapoptosis functions of miR-25, a conclusion that is consistent with previous work [39]. Overexpression of miR-25 by infection of cells with lenti-pre-miR-25 fully restored miR-25 levels and prevented the proapoptotic actions of corticosterone.

MOAP1 promotes caspase-dependent apoptosis by binding proapoptotic BAX via its Bcl-2 homology-3- (BH3-) like domain. MOAP1 is rapidly upregulated in response to multiple apoptotic stimuli [40, 46, 47]. Our findings are consistent with previous work that MOAP1 is a direct target for miR-25. We propose that miR-25-dependent downregulation of MOAP1 is the principal avenue of protection against corticosterone-induced injury of VSMCs. We also found that miR-25 overexpression conferred increased levels of protective Bcl2, whereas p70S6K was repressed, and the effects were reversed by antagomiR-25. p70S6K is a member of the IGF-1 and PI-3 kinase signaling pathways that include Akt and mTOR intermediates. Stimulation of these pathways usually confers a growth response but can also regulate apoptosis and autophagy by crosstalk with other pathways. In our study, we found that p70S6K phosphorylation was increased by treatment of VSMCs with corticosterone in parallel with increased ROS and decreased miR-25. Although causal roles for S6K1 in the regulation cannot yet be confirmed, such roles would be consistent with its activation by knockdown of miR-25 and repression by pre-miR (Figure 7(c)). A negative role of S6K1 in this pathway is also supported by the overriding effects of rapamycin on corticosterone-induced apoptosis.

## 4. Conclusion

We present novel observations that glucocorticoid treatment of VSMCs represses the expression of miR-25 while increasing ROS production, activity of S6K1, and apoptotic index. Overexpression of miR-25 reversed these effects. We propose that regulation of MOAP1 by miR-25 is the central mechanism and such signaling by corticosterone may be associated with ROS and S6K1. The study has direct implications to the established proatherogenic effects of psychological stresses including those associated with the occupational environment, which are known to be accompanied by increased levels of glucocorticoids. MicroRNA-25 is a potential therapeutic target that may ameliorate the plaque-destabilizing effects of glucocorticoid-induced apoptosis of VSMCs in advanced atherosclerosis disease.

### 4.1. Limitations of the Study

We acknowledge that these studies are limited to mouse VSMCs treated with the murine glucocorticoid hormone corticosterone and do not necessarily fully mimic human VSMCs treated with cortisol. The experiments are also limited to cultured cells and may not accurately reflect the actions or pathways driven by these glucocorticoid hormones in vivo. Therefore, additional experiments are required for the results to be considered relevant to humans or clinical pathologies.

## Data Availability

All the data used to support the findings of this study are available from the corresponding author upon request.
